# Correlation of Urinary Bisphenol A and Phthalates With Overweight and Obesity in Children

**DOI:** 10.7759/cureus.105308

**Published:** 2026-03-16

**Authors:** Shravani G U, Sunil Kumar B M, Manjunath P R, Vignesh Gadupudi

**Affiliations:** 1 Pediatrics, M. S. Ramaiah University of Applied Sciences, Bangalore, IND; 2 Endocrinology, M. S. Ramaiah University of Applied Sciences, Bangalore, IND; 3 Radiology, Sri Ramachandra Institute of Higher Education and Research, Chennai, IND

**Keywords:** bisphenol a, childhood obesity, endocrine disruptors, environmental exposure, metabolic risk, pediatric endocrinology, phthalates, urinary biomarkers

## Abstract

Background

Bisphenol A (BPA) and phthalates are endocrine-disrupting chemicals widely used in plastics, food packaging, and consumer goods. These compounds may contribute to childhood obesity by interfering with hormonal regulation and metabolic pathways.

Objective

This study aimed to evaluate urinary BPA and phthalate concentrations in normal-weight and overweight or obese children and to assess correlations with anthropometric measures.

Methods

A prospective observational study was conducted in the Department of Pediatrics, M. S. Ramaiah Medical College, Bangalore, India, from September 2023 to March 2025. Ninety children aged 2-18 years were enrolled: 45 overweight or obese (cases) and 45 age- and sex-matched normal-weight controls. Anthropometric parameters, including height, weight, body mass index, waist circumference, hip circumference, waist-to-hip ratio, and triceps skinfold thickness, were measured. Midstream urine samples were collected and analyzed for BPA and phthalates using a double-antibody sandwich enzyme-linked immunosorbent assay. Creatinine correction was performed. Statistical analysis included independent t-tests, chi-squared tests, and Pearson's correlation coefficients.

Results

Mean urinary BPA levels were significantly higher in overweight or obese children (4.59 ± 1.23 ng/mL) compared with controls (1.57 ± 0.48 ng/mL; t = 13.6; p < 0.001). Mean urinary phthalates were also elevated in cases (230.90 ± 35.30 ng/mL) versus controls (100.30 ± 18.70 ng/mL; t = 19.4; p < 0.001). Raw urinary BPA and phthalate levels demonstrated positive correlations with body mass index, waist circumference, and triceps skinfold thickness. Interestingly, creatinine-corrected BPA and phthalate levels were higher in cases.

Conclusion

Urinary BPA and phthalate concentrations were significantly associated with overweight and obesity in Indian children. These findings support the role of endocrine-disrupting chemicals as potential obesogens and highlight the need for public health strategies to reduce pediatric exposure to plastics.

## Introduction

Childhood obesity is a growing public health challenge, with rising prevalence worldwide and significant long-term health implications. Obese children are at increased risk for type 2 diabetes, cardiovascular disease, and early morbidity [1,2]. In India, rapid urbanization and lifestyle changes in recent decades have further contributed to pediatric obesity [3].

In addition to diet and physical activity, environmental exposures are increasingly recognized as important contributors. In children, the primary exposure to bisphenol A (BPA) in India occurs through dietary intake due to migration from plastic food containers, baby bottles, and epoxy-lined food packaging into infant formula, milk, and packaged foods. Additional exposure pathways include ingestion of contaminated drinking water, household dust, and dermal contact with BPA-containing consumer products such as toys and thermal paper, which may contribute to early-life endocrine disruption [4-7].

Several studies from Western countries have reported associations between urinary BPA or phthalate levels and obesity in children [8,9]. However, there is limited evidence from Indian pediatric populations. The objective of this study was to evaluate urinary BPA and phthalate concentrations in normal-weight and overweight or obese children and to assess their correlation with anthropometric measures.

## Materials and methods

Study design and setting

This prospective observational study was conducted in the Department of Pediatrics, M. S. Ramaiah Medical College, Bangalore, India, from September 2023 to March 2025.

Participants

A total of 90 children between two and 18 years of age were enrolled in the study. Forty-five children classified as overweight or obese were included as cases, while 45 age- and sex-matched normal-weight children served as controls. Nutritional status was determined using body mass index (BMI) for age according to the World Health Organization (WHO) growth reference standards. BMI was calculated as weight in kilograms divided by height in meters squared (kg/m²). Children with BMI-for-age between +1 and +2 standard deviations (SD) were classified as overweight, and those with BMI-for-age greater than +2 SD were classified as obese. Children with BMI-for-age between −2 SD and +1 SD were considered normal weight and were included in the control group [10].

Inclusion criteria

Children aged 2-18 years whose parents or legal guardians provided written informed consent were eligible for inclusion.

Exclusion criteria

Children with known endocrine or genetic causes of obesity, those receiving long-term medications affecting body weight (such as corticosteroids or valproate), or those with chronic illnesses were excluded.

Anthropometric measurements

Height was measured using a stadiometer, and weight was recorded using a calibrated digital weighing scale. BMI was calculated as weight in kilograms divided by height in meters squared. Waist and hip circumferences were measured with a non-stretchable measuring tape, and the waist-to-hip ratio was derived. Triceps skinfold thickness was measured using Harpenden calipers. All measurements were taken twice, and the average values were used for analysis.

Assessment of dietary habits and physical activity

Information regarding dietary habits and physical activity was obtained using a structured questionnaire administered to parents or guardians and older children during the clinical visit. Dietary behavior was assessed by recording the frequency of junk food consumption per week, categorized as once, twice, thrice, four times, five times, six times, or everyday consumption. Physical activity levels were assessed based on self-reported daily activity duration, categorized as no activity, half an hour, one hour, or two hours of daily physical activity. These variables were analyzed using the chi-squared test to determine associations between lifestyle factors and overweight or obesity.

Urine sample collection and analysis

Midstream urine samples were collected in sterile containers from each participant, immediately stored at 4°C, and processed. Urinary levels of BPA and phthalates were quantified using commercially available enzyme-linked immunosorbent assay (ELISA) kits according to the manufacturer's instructions (Elabscience Biotechnology Inc., Houston, TX, USA). To account for urinary dilution, concentrations were normalized to urinary creatinine levels.

Statistical analysis

All data were entered into Microsoft Excel (Microsoft Corp., Redmond, WA, USA) and analyzed using IBM SPSS Statistics for Windows, V. 26.0 (IBM Corp., Armonk, NY, USA). Continuous variables were expressed as mean ± SD. Independent t-tests were used to compare group means. Pearson's correlation coefficients were calculated to evaluate relationships between urinary BPA or phthalate concentrations and anthropometric variables. A p-value of <0.05 was considered statistically significant.

Ethics approval

This study was reviewed and approved by the Institutional Ethics Committee of M. S. Ramaiah Medical College (approval number: MSRMC/EC/PG-58/04-2023). Written informed consent was obtained from the parents or legal guardians of all participants prior to inclusion.

## Results

Participant characteristics

A total of 90 children were included in the study, consisting of 45 overweight or obese children (cases) and 45 age- and sex-matched normal-weight children (controls). The mean age of cases was 11.6 ± 3.4 years, while that of controls was 10.9 ± 3.1 years. Males comprised 58% of cases and 53% of controls. Anthropometric characteristics, including BMI, waist circumference, hip circumference, waist-to-hip ratio, and triceps skinfold thickness, were significantly higher in cases compared with controls.

Table 1 presents the age-wise distribution of the study participants. The sample was evenly divided across three age groups (3-7 years, 8-12 years, and 13-17 years), with 30 children in each group, totaling 90 participants.

**Table 1:** Age-wise distribution of the study participants N: number; %: percentage

Age group (in years)	N	%
3-7	30	33.3
8-12	30	33.3
13-17	30	33.3

Gender distribution

Table 2 shows the gender distribution of the study participants. In the overweight or obese group, 75.6% were male, and 24.4% were female, whereas in the normal-weight group, 55.6% were male, and 44.4% were female.

**Table 2:** Gender distribution among the study participants

Gender	Overweight/obese		Normal weight	
	Frequency	Percentage	Frequency	Percentage
Male	34	75.6	25	55.6
Female	11	24.4	20	44.4

Mean weight

Table 3 presents the mean body weight of the study participants. The overweight or obese group had a higher mean body weight (63.04 ± 20.15 kg) compared with the normal-weight group (27.72 ± 14.35 kg). Because the study population included children across a wide age range (3-17 years), BMI for age based on WHO growth reference standards was used as the primary criterion for the classification of overweight and obesity. Therefore, BMI and other anthropometric indicators were considered more appropriate measures for intergroup comparison than absolute body weight.

**Table 3:** Mean and SD of weight among the study participants SD: standard deviation; P-value: probability value; kg: kilograms; t-value: Student's t-test statistic

Variable	Group	Mean (kg)	SD	t-value	P-value
Weight	Overweight/obese	63.04	20.15	3.345	0.05
	Normal weight	27.72	14.35		

Table 4 shows the mean BMI of the study participants. The overweight or obese group had a mean BMI of 26.53, compared with 16.13 in the normal-weight group.

**Table 4:** Mean and standard deviation of BMI among the study participants t-value: Student's t-test statistic; P-value: probability value; BMI: body mass index

Variables	Group	Mean	Standard deviation	t-value	P-value
BMI	Overweight/obese	26.53	4.028	5.507	0.02
	Normal weight	16.13	2.760		

Table 5 presents the mean waist-to-hip ratio of the study participants. The overweight or obese group had a mean waist-to-hip ratio of 0.94, compared with 0.82 in the normal-weight group.

**Table 5:** Mean and standard deviation of the waist-to-hip ratio among the study participants t-value: Student's t-test statistic; P-value: probability value

Variables	Group	Mean	Standard deviation	t-value	P-value
Waist-to-hip ratio	Overweight/obese	0.9395	0.02952	4.446	0.03
	Normal weight	0.8208	0.03685		

Table 6 summarizes the triceps skinfold thickness among the study participants. The overweight or obese group had a mean thickness of 19.82 mm, compared with 11.09 mm in the normal-weight group.

**Table 6:** Mean and standard deviation of triceps skinfold thickness among the study participants t-value: Student's t-test statistic; P-value: probability value

Variables	Group	Mean	Standard deviation	t-value	P-value
Triceps skinfold thickness	Overweight/obese	19.82	4.711	7.569	0.007
	Normal weight	11.09	6.484		

Table 7 presents the mean urinary BPA concentrations (ng/mL) among the study participants. Levels were higher in the overweight or obese group compared with the normal-weight group.

**Table 7:** Mean and standard deviation of BPA among the study participants t-value: Student's t-test statistic; P-value: probability value; BPA: bisphenol A

Variables	Group	Mean	Standard deviation	t-value	P-value
BPA	Overweight/obese	4.588	1.2311	19.153	0.001
	Normal weight	1.567	0.4807		

Table 8 shows the mean urinary phthalate concentrations (ng/mL) among the study participants. Levels were higher in the overweight or obese group compared with the normal-weight group.

**Table 8:** Mean and standard deviation of phthalates among the study participants t-value: Student's t-test statistic; P-value: probability value

Variables	Group	Mean	Standard deviation	t-value	P-value
Phthalates	Overweight/obese	230.97	35.328	16.116	0.001
	Normal weight	100.37	18.702		

Table 9 displays a comparison between corrected urinary BPA levels in normal-weight and overweight children.

**Table 9:** Mean and standard deviation of corrected BPA among the study participants t-value: Student's t-test statistic; P-value: probability value; BPA: bisphenol A

Variables	Group	Mean	Standard deviation	t-value	P-value
Corrected BPA	Overweight/obese	4.948	0.7771	72.663	0.001
	Normal weight	8.187	3.7595		

Table 10 displays a comparison between corrected phthalate levels in normal-weight and overweight children.

**Table 10:** Mean and standard deviation of corrected urinary phthalates among the study participants t-value: Student's t-test statistic; P-value: probability value

Variables	Group	Mean	Standard deviation	t-value	P-value
Corrected phthalates	Overweight/obese	0.050	0.0135	75.916	0.001
	Normal weight	0.116	0.0768		

Lifestyle factors: dietary habits and physical activity

Dietary habits and physical activity patterns were also evaluated among the study participants. Dietary behavior was assessed based on the frequency of junk food consumption per week. A higher proportion of overweight or obese children reported more frequent junk food intake compared with normal-weight children. However, the difference between the two groups was not statistically significant.

Physical activity levels were assessed based on the average duration of daily activity. A greater proportion of overweight or obese children reported lower levels of daily physical activity compared with normal-weight children. Although overweight or obese children tended to be less physically active, the difference between the groups did not reach statistical significance.

These findings indicate that overweight or obese children in this study generally reported higher junk food consumption and lower levels of physical activity compared with controls. Table 11 displays the distribution of dietary habits and physical activity among the study participants.

**Table 11:** Distribution of dietary habits and physical activity among the study participants N: number of participants; %: percentage; P-value: probability value

Variable	Category	Overweight/obese (N = 45)	Normal weight (N = 45)	P-value
Junk food consumption per week	≤2 times/week	38 (84.4%)	45 (100%)	0.502
	≥3 times/week	7 (15.6%)	0 (0%)	
Daily physical activity	No activity	10 (22.2%)	5 (11.1%)	0.140
	30 minutes/day	15 (33.3%)	12 (26.7%)	
	1 hour/day	15 (33.3%)	21 (46.7%)	
	2 hours/day	5 (11.1%)	7 (15.6%)	

## Discussion

The present study demonstrated that urinary BPA and phthalate concentrations were significantly associated with overweight and obesity in children. These findings are consistent with growing evidence that endocrine-disrupting chemicals (EDCs) may play a role in the pathogenesis of pediatric obesity [11,12].

Age-related associations

Overweight and obese participants in this study were more frequently represented in older age groups, supporting the hypothesis that prolonged or cumulative exposure may strengthen obesogenic effects. Similar observations were reported in the US National Health and Nutrition Examination Survey (NHANES), where higher urinary BPA levels were associated with obesity in children and adolescents aged 6-19 years [11]. Hatch et al. [12] also found that phthalate metabolites correlated with increased BMI and waist circumference, particularly in boys aged 8-14 years. In contrast, studies in preschool-aged children reported weaker or absent associations [13], suggesting that the metabolic effects of EDCs may emerge later in childhood, potentially in relation to puberty.

Gender differences

This study observed a male predominance in the overweight and obese group. Previous research has also identified stronger associations between phthalates and adiposity among boys [12]. Boas et al. [14] suggested that boys may be more sensitive to EDCs due to interference with androgen signaling. Conversely, Harley et al. [15] reported higher BMI in girls exposed to phthalates prenatally, and Valvi et al. [16] found similar associations between early-life exposures and adiposity in girls. These inconsistencies highlight the possibility of sex-specific biological responses and behavioral factors influencing exposure.

Anthropometric findings

Differences in body composition between groups, including BMI, waist circumference, hip circumference, waist-to-hip ratio, and triceps skinfold thickness, align with earlier studies linking EDCs to central and peripheral fat accumulation. Waist circumference, in particular, has been emphasized as a predictor of cardiometabolic risk in children [17], while waist-to-hip ratio has been proposed as a marker for visceral adiposity and insulin resistance [18]. Our findings are also in line with Freedman et al. [19], who demonstrated that skinfold thickness strongly correlates with total body fat. However, other studies, such as Wolff et al. [20], did not identify significant associations between phthalates and adiposity in younger children, reinforcing the importance of exposure timing.

Lifestyle factors

Dietary habits and physical activity were assessed using a structured questionnaire. Overweight and obese children in the present study tended to report higher junk food consumption and lower levels of daily physical activity compared with normal-weight children, although the differences were not statistically significant. Prior research supports these observations: irregular eating patterns and frequent consumption of processed foods have been linked with obesity [21,22], while sedentary lifestyles are strongly associated with higher BMI [23]. The role of EDCs may be compounded by these behavioral factors, producing additive effects on adiposity.

Urinary BPA and phthalate concentrations

The significantly higher urinary BPA and phthalate concentrations in overweight and obese participants align with prior studies [24]. Creatinine-corrected analyses demonstrated differing trends between the groups. The mean creatinine-corrected BPA level was 4.95 ± 0.78 in overweight or obese children compared with 8.19 ± 3.76 in normal-weight controls, while creatinine-corrected phthalate levels were 0.050 ± 0.013 in cases and 0.116 ± 0.077 in controls. These findings suggest that creatinine adjustment may influence the interpretation of urinary biomarker concentrations in pediatric populations. Similar findings have been noted in pediatric studies where hydration status and renal function influenced creatinine-based adjustments [25]. This highlights the limitations of creatinine correction in children and suggests that raw values may better represent exposure in this age group.

Strengths and limitations

Key strengths of this study include being one of the first from India to evaluate urinary BPA and phthalates in relation to childhood obesity, the use of validated ELISA methods for detection, and standardized anthropometric assessments. Limitations include the modest sample size, reliance on single-spot urine samples, and absence of detailed dietary and socioeconomic data. The cross-sectional design limits causal inference.

Implications

These findings underscore the importance of considering environmental exposures in pediatric obesity prevention strategies. Reducing the use of plastics and processed food packaging may help mitigate risk, particularly among older children. Longitudinal studies with larger cohorts are needed to confirm causality, evaluate sex-specific susceptibilities, and inform public health policy.

## Conclusions

This study demonstrated that urinary concentrations of BPA and phthalates were significantly higher in overweight and obese children compared with their normal-weight peers. Elevated levels of these EDCs were positively associated with increased BMI, waist circumference, and triceps skinfold thickness, suggesting their potential contribution to adiposity. Behavioral factors such as greater meal frequency and lower levels of physical activity were also more common among overweight and obese children, reinforcing the multifactorial nature of pediatric obesity.

Taken together, these findings indicate that environmental exposure to EDCs may influence the development of obesity in children, particularly during vulnerable developmental stages such as puberty. Preventive strategies should not only emphasize lifestyle modification but also incorporate measures to reduce exposure to plastics and processed food packaging. Larger, longitudinal studies are warranted to confirm causality and to guide public health interventions aimed at mitigating the impact of environmental chemicals on child health.
